# New Variant With a Previously Unrecognized Mechanism of Pathogenicity in Hypertrophic Cardiomyopathy

**DOI:** 10.1161/CIRCULATIONAHA.120.048295

**Published:** 2021-08-31

**Authors:** Yasmine Aguib, Mona Allouba, Roddy Walsh, Ayman M. Ibrahim, Sarah Halawa, Alaa Afify, Mohammed Hosny, Pantazis I. Theotokis, Aya Galal, Sara Elshorbagy, Mohamed Roshdy, Heba S. Kassem, Amany Ellithy, Rachel Buchan, Nicola Whiffin, Shehab Anwer, Stuart A. Cook, Ahmed Moustafa, Ahmed ElGuindy, James S. Ware, Paul J.R. Barton, Magdi Yacoub

**Affiliations:** 1 Aswan Heart Centre, Egypt (Y.A., M.A., A.M.I., S.H., A.A., M.H., A.G., S.E., M.R., A.E., S.A., A. ElGuindy, M.Y.).; 2 National Heart and Lung Institute, Imperial College London, United Kingdom (Y.A., M.A., P.I.T., R.B., N.W., A. ElGuindy, J.S.W., P.J.R.B., M.Y.).; 3 Department of Experimental Cardiology, Amsterdam UMC, the Netherlands (R.W.).; 4 Faculty of Science, Cairo University, Egypt (A.M.I.).; 5 Biotechnology Graduate Program (S.H., A.M.), American University in Cairo, New Cairo, Egypt.; 6 Department of Biology (A.M.), American University in Cairo, New Cairo, Egypt.; 7 Cardiology Department, Faculty of Medicine, Cairo University, Egypt (M.H.).; 8 Clinical Genomics Center, Alexandria Faculty of Medicine, Egypt (H.S.K.).; 9 Royal Brompton and Harefield Hospitals, London, United Kingdom (R.B., N.W., J.S.W., P.J.R.B.).; 10 Wellcome Centre for Human Genetics, University of Oxford, United Kingdom (N.W.).; 11 Duke–National University of Singapore Medical School (S.A.C.).; 12 National Heart Centre Singapore (S.A.C.).; 13 MRC London Institute of Medical Sciences, United Kingdom (S.A.C., J.S.W., P.J.R.B.).; 14 Harefield Heart Science Centre, United Kingdom (M.Y.).

**Keywords:** cardiomyopathy, hypertrophic, codon, nonsense, frameshift mutation, genetic variation, high-throughput nucleotide sequencing

Hypertrophic cardiomyopathy (HCM) represents one of the most common inherited cardiac conditions. It is genetically and clinically heterogeneous and is most commonly caused by variants in sarcomere-encoding genes, including *MYH7*, which represents the second most common cause of familial HCM.^1^ Missense variants in *MYH7* are believed to cause HCM through gain-of-function actions: Variants produce an abnormal activated protein that incorporates into the sarcomere as a poison peptide.^2^ Haploinsufficiency in *MYH7* is not a recognized disease mechanism for HCM, and heterozygous variants that introduce premature termination codons (PTCs; ie, nonsense, frameshift, and splice variants) have not previously been demonstrated to be associated with this disease.^1^

Here, we report the frameshift variant c.5769delG in *MYH7* (ENST00000355349) that is associated with HCM in a large series of unrelated Egyptian patients with HCM assessed at the Aswan Heart Center. All patients gave written informed consent, and the study was reviewed and approved by the institutional research ethics committee (FWA00019142; Research Ethics Committee code 20130405MYFAHC_CMR_20130330). c.5769delG was found in 3.3% of patients (17 of 514) yet was absent from locally recruited ethnically matched controls (n=400, *P*_Fisher_=5.0×10^−^^5^), the Genome Aggregation Database (n=125,748, *P*_Fisher_=2.0×10^−^^41^), and the Iranome reference database (n=800, *P*_Fisher_=9.98×10^−^^8^). The variant was identified in 1 individual from the Great Middle Eastern population study^3^ (n=993, *P*_Fisher_=1.2×10^−7^), but the cardiac phenotype is not known for this subject. The variant reported here has not previously been seen in >6000 predominantly White patients with HCM.^4^

Detailed study of a large family showed the cosegregation of c.5769delG with HCM (logarithm of the odds score, 3.01), and no other rare protein-altering variants were found in other established HCM genes.^4^ The proband carrying the frameshift variant presented with the disease at 29 years of age, and all affected family members carried the variant (Figure [A], left). Proband and family members were clinically evaluated by cardiologists experienced in diagnosing and managing patients with HCM who were initially blinded to the genotype of each individual (Figure [A], left and Figure [B]). The diagnosis of HCM was established according to the 2014 European Society of Cardiology HCM guidelines: individuals with wall thickness ≥15 mm involving ≥1 segments of the left ventricle (using transthoracic echocardiography) that cannot be explained by loading conditions. In addition, first-degree family members of patients with confirmed HCM were diagnosed if the wall thickness of ≥1 left ventricular segments was ≥13 mm in the absence of an alternative explanation. In patients with borderline increased wall thickness (13–14 mm), other features suggestive of HCM were evaluated, including diastolic function, left atrial dimensions, systolic anterior motion of the mitral valve, dynamic left ventricular outflow tract obstruction, and late gadolinium enhancement on cardiac magnetic resonance imaging (Figure [B]). In total, 16 members of the family were evaluated, with 6 patients being diagnosed with HCM. Histopathological characterization of patients’ tissues shows high levels of interstitial fibrosis, elevated transforming growth factor-β1 expression, and a significant increase in myocyte diameter characteristic of HCM (Figure [C]).

**Figure.**
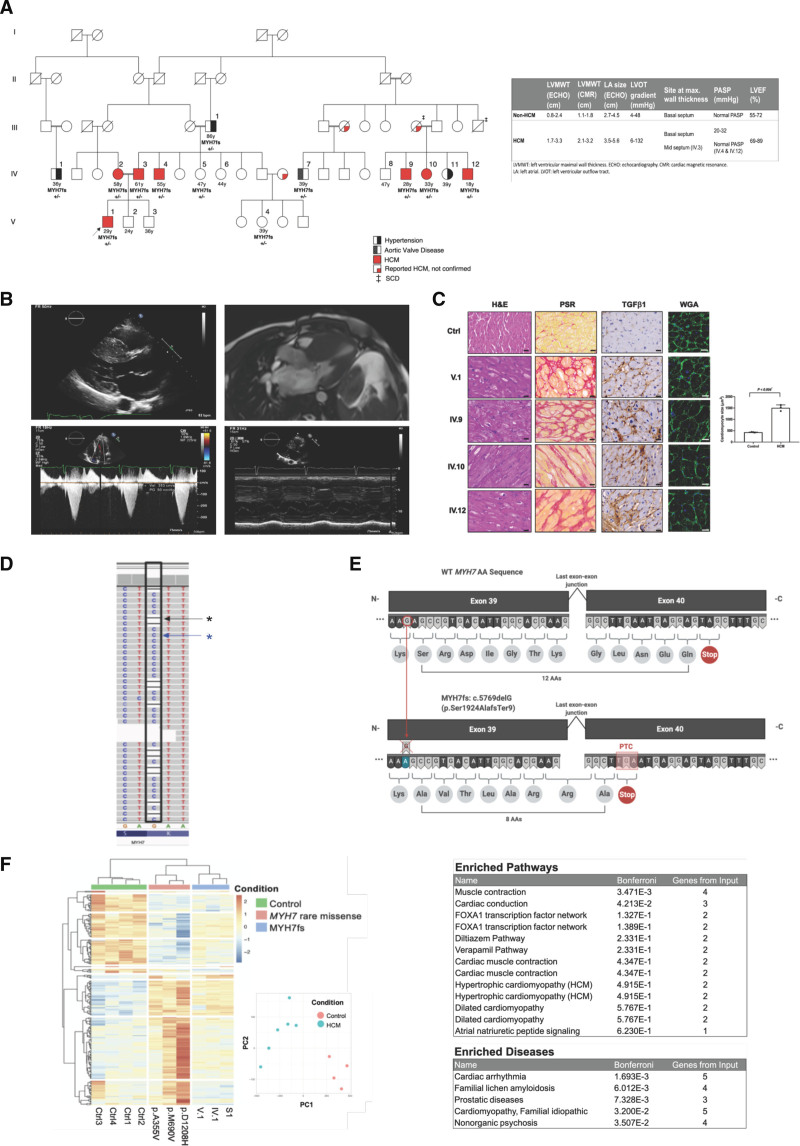
**A frameshift variant in *MYH7* (c.5769delG) is associated with HCM. A**, **Left**, *MYH7* (c.5769delG) segregates with hypertrophic cardiomyopathy (HCM) in a large Egyptian family (limit of detection, 3.01). The proband is marked with an arrow. Ages of family members at diagnosis are shown. The variant segregated in all affected family members. **Right**, Clinical and genetic characteristics of the proband and affected family members depicted in the pedigree. Clinical history at presentation for patients with HCM. V.1: dyspnea, New York Heart Association (NYHA) class III, palpitations, and myectomy; IV.2: dyspnea, NYHA class II, and palpitations; IV.3: dyspnea, NYHA class II, and presyncope; IV.4: dyspnea, NYHA class III, and angina; IV.9: dyspnea, NYHA class II, paroxysmal nocturnal dyspnea, and myectomy; IV.10: dyspnea, NYHA class II, angina, palpitations, and myectomy; and IV.12: dyspnea, NYHA class III, angina, palpitations, and myectomy. Cardiac magnetic resonance (CMR) data were not available for V.2, V.4, IV.6, II.1, IV.2, and IV.3. **B**, Proband (ie, V.1 in pedigree) showing classic HCM features assessed by echocardiography and CMR. **Top left** and **top right**, Severe asymmetrical septal hypertrophy (basal interventricular septum measuring 36 mm) with systolic anterior motion of the mitral valve (**bottom left**) causing a peak systolic gradient of 50 mm Hg across the left ventricular outflow tract at rest (**bottom right**). **C**, **Left**, Histological characterization of HCM myectomy tissues (proband: V.1 and affected family members IV.9, IV.10, IV.12) compared with control myocardial tissues, stained with hematoxylin and eosin (H&E), picrosirius red (PSR), transforming growth factor-β1 (TGFβ1) immunohistochemistry, and wheat germ agglutinin (WGA). Scale bars are 20 µm. **Right**, WGA quantification in control subjects vs patients with HCM (*P*_Fisher_<0.004, unpaired *t* test). **D**, RNA sequencing analysis of human heart tissue from the proband confirms the presence of both wild-type (WT; blue asterisk) and mutant (black asterisk) transcripts. *MYH7* is on the reverse strand. **E**, Schematic of the *MYH7* frameshift variant (*MYH7fs*) is predicted to lead to expression and translation of a truncated peptide. The cDNA sequence and predicted resulting amino acid sequence are shown for WT (**top**) and variant *MYH7* (**bottom**) alleles. The frameshift variant (deletion of nucleotide C in exon 39) is predicted to result in an nonsense mediated decay–incompetent premature termination codon downstream of the last exon-exon junction. **F**, **Left**, Differentially expressed genes in RNA sequencing in left ventricular tissue from patients with *MYH7fs*, *MYH7* rare missense variants (p.A355V, p.M690V, and p.D1208H), and control subjects. Each row and column in the heat map represent a differentially expressed gene and sample. Samples and genes are clustered according to the correlation among the genes and among the samples, respectively. The expression values are color-coded and scaled for each gene (ie, by row). Principal component (PC) analysis of gene signature of patients with HCM with the c.5769delG variant, patients with HCM with different rare missense *MYH7* variants, and control subjects (inset). **Right**, Enrichment analysis of differentially expressed Gene Ontology terms in patients with HCM with the c.5769delG variant and controls. Enriched pathways (**top**) and diseases (**bottom**) that were downregulated in samples with *MYH7* c.5769delG compared with controls.

RNA sequencing of myocardial tissue (obtained during surgical myectomy) from the proband carrying c.5769delG confirmed expression of the variant *MYH7* transcript (Figure [D]). The predicted sequence of the variant *MYH7* transcript shows that the PTC resides downstream of the last exon-exon junction (Figure [E]). PTCs in the last exon of a gene, or in the last 50 to 55 bp of the penultimate exon, generally escape nonsense mediated decay, a posttranscriptional mechanism that degrades mRNAs harboring a PTC.^5^ The translated protein sequence would produce an alternate c-terminal sequence just 4 amino acids shorter than the wild-type protein.

We then investigated the gene expression profiles of patients with HCM carrying the *MYH7* frameshift variant (n=3), patients with HCM carrying different *MYH7* variants (rare missense; n=3), and control subjects (n=4). The analysis identified 254 differentially expressed genes (Bonferroni-corrected *P*_Fisher_<0.05) across the 3 groups with the *MYH7* frameshift variant clustering with HCM (Figure [F], left). Principal component analysis shows distinct clustering of patients with HCM (both groups) compared with control subjects (Figure [F], left). Enrichment analysis for differentially expressed genes between patients with the *MYH7* frameshift variant and control subjects showed pathways related to muscle contraction, cardiac conduction, and dilated/hypertrophic cardiomyopathy, consistent with the molecular changes characteristic of HCM (Figure [F], right).

In *MYH7*, only missense variants have previously been proven to cause HCM. Here, we provide the first evidence of an association with an nonsense mediated decay–incompetent frameshift variant, implicating a new class of genetic variants in disease. Although heterozygous null alleles, including nonsense mediated decay–competent PTCs, would not be considered pathogenic for HCM in isolation (but could be potent modifiers if found in trans with a disease allele or pathogenic if homozygous), distal PTCs may lead to expression of an abnormal protein that may be disease causing.

In conclusion, this study identifies a new class of pathogenic variants in HCM with important clinical implications and confirms the value of studying diverse populations.

Data, analytical methods, and study materials will be made available to other researchers through direct communication.

## Sources of Funding

This study is part of the Egyptian Collaborative Cardiac Genomics Project. It was supported by the Science and Technology Development Fund government grant (Egypt), the Wellcome Trust (107469/Z/15/Z; 200990/A/16/Z), the Medical Research Council (United Kingdom), the NIHR Royal Brompton Cardiovascular Biomedical Research Unit, the NIHR Imperial College Biomedical Research Center, and a Health Innovation Challenge Fund award from the Wellcome Trust and Department of Health, United Kingdom (HICF-R6–373). Drs Allouba and Halawa are funded by Al Alfi Foundation to support their PhD degrees at Imperial College London and American University in Cairo, respectively. Dr Aguib is supported by Fondation Leducq (11 CVD-01). Dr Whiffin is supported by a Rosetrees and Stoneygate Imperial College Research Fellowship. Dr Walsh is supported by an Amsterdam Cardiovascular Science Fellowship.

## Disclosures

None.
